# Role of Macrophages in the Progression and Regression of Vascular Calcification

**DOI:** 10.3389/fphar.2020.00661

**Published:** 2020-05-08

**Authors:** Yalan Li, Zhen Sun, Lili Zhang, Jinchuan Yan, Chen Shao, Lele Jing, Lihua Li, Zhongqun Wang

**Affiliations:** ^1^Department of Cardiology, Affiliated Hospital of Jiangsu University, Zhenjiang, China; ^2^Department of Pathology, Affiliated Hospital of Jiangsu University, Zhenjiang, China

**Keywords:** vascular calcification, macrophages, polarization drift, phenotypic transformation, inflammation, matrix vesicles

## Abstract

Vascular calcification is an abnormal cell-mediated process in which bone-specific hydroxyapatite crystals are actively deposited on the blood vessel wall and is a significant pathological basis for the increased incidence and mortality of adverse cardiovascular events. Macrophages play an important regulatory role in the occurrence, development, and regression of vascular calcification. After the tissue microenvironment changes, macrophages subsequently change their polarity and phenotype or secrete functional substances as an adaptive response. As research on macrophages continue to move into this field, we gain a new understanding of the mechanism of the formation and regression of vascular calcification, which might offer valuable new intervention targets for the prevention and inhibition of vascular calcification. This review summarizes a wealth of research in this field and explores the roles of macrophages in the development process of vascular calcification.

## Introduction

Vascular calcification is a prevalent complication of various diseases that increases the incidence of acute coronary cardiovascular events and seriously impacts the quality of life of patients due to increased vascular stiffness and decreased compliance (Stabley and Towler, 2017; Wyatt and Drueke, 2017; Georgianos et al., 2018). Currently, the underlying mechanisms that explain the formation of vascular calcification can be summarized as the induction of osteogenesis, weakening of mineralization inhibition, formation of a nucleation complex, and cell apoptosis (Giachelli, 2004). Macrophages are considered a double-edged sword in the process of vascular calcification. Macrophages can promote vascular calcification by releasing inflammatory factors and extracellular vesicles but macrophages can also suppress vascular calcification by secreting anti-inflammatory factors and differentiating into osteoclast-like cells. However, the specific mechanism by which macrophages influence the formation and regression of vascular calcification has not been fully elucidated. In this work, the authors integrate a series of studies by relevant scholars to explore the exact mechanism of the role of macrophages in formation and regression of vascular calcification and offer new ideas for prevention and treatment of vascular calcification and bone metabolic diseases.

## Characteristics of Vascular Calcification

### Concept and Progression of Vascular Calcification

Vascular calcification was previously considered to be the passive deposition of calcium and phosphorus in the vascular wall after a long term metabolic imbalance and to not be regulated by other factors. At the end of the 20th century, Demer proposed the concept of high ossification of vascular calcification. Currently, it is increasingly recognized that vascular calcification is an active and highly regulatable biological process that shares certain similarities with bone formation (Sage et al., 2010; Wu et al., 2013). Vascular calcification is positively correlated with age and is an independent risk factor for increased mortality and morbidity of cardiovascular diseases, and it usually occurs in atherosclerosis, end-stage renal disease, diabetes, or even rare genetic diseases (Janssen, 2017; Stabley and Towler, 2017; Wyatt and Drueke, 2017). According to the location of occurrence, vascular calcification can be divided into intimal calcification, medial calcification (Monckeberg’s sclerosis), valvular calcification, and calcification prevention (Shao et al., 2016). Intimal calcification often occurs in an atherosclerotic plaque, which is related to macrophages and smooth muscle cells in the lipid-rich area within lesions. Plaque calcification displays a more scattered and patchy distribution, which can easily lead to plaque instability and ischemic events. Medial calcification has a continuous and linear distribution and correlates well with phenotypic de-differentiation of vascular smooth muscle cells (Villa-Bellosta et al., 2016). In addition, calcification results in completely different clinical manifestations and outcomes according to its morphology, and microcalcification contributes to plaque instability, whereas macrocalcification makes plaques more prone to stabilization (Yahagi et al., 2017).

The pathogenesis of vascular calcification is complex and manifold, including high calcium and phosphorus levels, a chondrogenic phenotype differentiated from vascular wall cells, decreased levels of natural calcification inhibitors (fetoglobulin A, pyrophosphate, osteopontin, matrix gla protein, etc.), inflammatory stimulation, apoptosis, and release of pro-calcified particles (New and Aikawa, 2011; Wu et al., 2013; Chen and Moe, 2015). The crucial step in the formation of vascular calcification is infiltration of monocytes/macrophages into the sub-endothelium of the aorta. Macrophages can promote vascular calcification through diverse mechanisms, such as the release of reactive oxygen species, pro-inflammatory cytokines, and matrix vesicles (MVs). The role of macrophages in the formation of vascular calcification should not be underestimated.

### Regression of Vascular Calcification

Vascular calcification shares many similarities with skeletal mineralization. Osteoblasts and osteoclasts maintain a dynamic balance during physiological bone remodeling. Osteoblasts deposit bone matrix proteins that can subsequently become mineralized and coordinate with bone resorption by activated osteoclasts. Once the dynamic balance between osteogenesis and osteolysis is broken, vascular calcification or pathological bone remodeling (such as osteoporosis) can occur (Yamanouchi et al., 2012).

In osteoporosis, the reduction of bone mineral formation is primarily related to the excessive activity of osteoclasts. In this process, the rate of bone resorption by osteoclasts often exceeds the rate of bone formation by osteoblasts. Osteoclasts are the cells exclusively responsible for degradation of bone tissue by secreting mineral-dissolving acids and proteases that degrade the organic matrix (Novack and Teitelbaum, 2008). Osteoclast-like cells are defined as multi-nucleated giant cells that exist outside of bone and are positive for tartrate resistant acid phosphate (TRAP) staining. These cells have the same morphological and histological characteristics as osteoclasts. Osteoclast-like cells have been verified to exist in calcified vascular tissue (Takeshita et al., 2000). The regression of vascular calcification may involve active osteoblast-like bone resorption activity. Chartrisa et al. found that macrophage-derived osteoclast-like cells reduced the calcified elastin mineral content by 80% *in vitro* without altering the integrity of elastin, which is associated with almost all types of vascular calcification. These results showed that osteoclast-like cells can effectively exert their de-mineralization ability outside the bone tissue (Simpson et al., 2007). In addition, high-dose bisphosphonates can also inhibit calcification by blocking the conversion of amorphous calcium phosphate into hydroxyapatite. Recent findings suggest that FYB-931 (a novel bisphosphonate compound) has selected additional effects independent of those of etidronate. In vitamin D3-treated rats, FYB-931 more potently inhibited aortic calcification and tartrate-resistant acid phosphatase (TRACP) activity than etidronate. Additionally, FYB-931 could cause a decrease in the serum levels of phosphorus and fibroblast growth factor 23 in a dose-dependent manner, which could promote inhibition of calcification (Ishida et al., 2019). Moreover, SNF472 is currently under development as an anti-calcifying agent. Ferrer et al. developed a novel and rapid *in vitro* spectrophotometric assay to prove that SNF472 is more powerful than bisphosphonates and pyrophosphate as a potential treatment for prevention of vascular calcification (Ferrer et al., 2017). The regulatory mechanisms of MGP, BMP-7, and fetoglobulin in promoting the regression of calcification still require further extensive research. Interventions targeting risk factors and regulators and inducing the formation of osteoclast-like cells may play a pivotal role in the regression of vascular calcification.

## Characteristics of Macrophages

### Definition

In the 1980s, using mouse models, M. Naito and K. Takahashi proposed that large cells with a macrophage morphology exist in the yolk sac blood island of E9 and the fetal liver of E10 and are immature and lack of phagocytic activity (Naito et al., 1990). This work gradually introduced macrophages into the public view and become a research hotspot in various fields. Macrophages are heterogeneous immune cells composed of multiple subsets that can effectively adapt to the microenvironment by altering their phenotype and physiological characteristics. After activation, macrophages can produce many growth stimulating factors, proteolytic enzymes, and pro-inflammatory factors and play an important role in inflammation, host defense, and tissue homeostasis (Sica et al., 2015).

It was previously thought that macrophages were formed by monocytes recruited from peripheral blood to tissues. However, recent studies have shown that macrophages in various tissues and organs have a heterogeneous origin and can be divided into yolk sac-derived macrophages (YSDM) and bone marrow derived macrophages (BMDM). YSDM arise from macrophage precursor cells of the yolk sac that migrate through blood vessels to organs and tissues during embryonic development, whereas BMDM are formed by differentiation of monocytes into individual tissues and organs in the peripheral blood after birth (Schulz et al., 2012). Macrophages are present in almost all tissues and have tissue specificity, such as Kupffer cells in liver tissue, alveolar macrophages in lung tissue, microglia cells in brain tissue, osteoclasts in bone tissue, Langerhans cells in the epidermis, and peritoneal macrophages in the abdominal cavity (Elisa and Frederic, 2016). These tissue-specific cells with different chromatin profiles also require specific growth factors and different transcription factors for differentiation and maintenance.

### Plasticity of Macrophages

**Figure 1:**
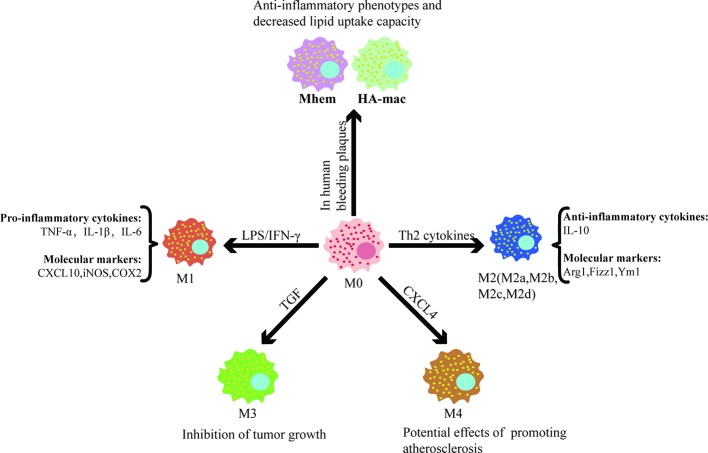
Different factors control macrophage polarization and the functions of different subsets. Different subsets of macrophages are shown with selected factors linked to their development. It is worthwhile to note that after polarization, macrophages have different functions and play a significant role in cardiovascular disease.

Remarkable plasticity and functional heterogeneity are important features of macrophages. Macrophage plasticity refers to the transformation of the function of macrophages in a specific direction and the shift of macrophages into different subsets or phenotypes congruent with the alteration of the microenvironment (**Figure 1**). This process is affected by different regulatory mechanisms, including intracellular signaling pathways, transcription factors, and epigenetic and post-translational modification.

Currently, the two most studied subsets are “the classical activated macrophage” (M1) and “the alternative activated macrophage” (M2) (Murray, 2017). Bacterial lipopolysaccharides and interferon-γ (INF-γ) are potent activators of macrophages that display the M1 phenotype, which is characterized by the secretion of pro-inflammatory cytokines, reactive oxygen species, and reactive nitrogen groups. Upon stimulation with Th2 cytokines, macrophages obtain an anti-inflammatory M2 phenotype and play an important role in parasitic infections resistance, tissue repair and remodeling, lipid metabolism, allergic reactions, and tumor progression (Braga et al., 2015; Porta et al., 2015; Sica et al., 2015; Bashir et al., 2016). Common M1 molecular markers include nitric oxide synthase (iNOS), cyclooxygenase 2 (COX2), CXC chemokines ligand 10 (CXCL10), etc. The M2 marker genes included arginase-1 (Arg1), found in inflammatory zone 1 (Fizz1), chitinase 3-like 3 (Ym1), etc. It is important to detect the molecular markers specifically expressed by macrophages of different plasticity to distinguish specific subsets. A large amount of data show that the functional status of macrophages follows a continuous change during the adaptive response to the external environment. The M1/M2 system cannot reflect the complexity of the macrophage subsets, and intermediate phenotypes must exist between them. M2 can be further divided into four subsets, namely M2a, M2b, M2c, and M2d, under different stimuli (Murray et al., 2014). TGF stimulates macrophages to differentiate into M3, which has the ability to suppress tumor growth (Kalish et al., 2017). CXCL4, a platelet-derived chemokine, is a potent stimulus that promotes macrophage polarization into the M4 phenotype, which has reduced phagocytosis and significantly downregulates CD163 expression (Gleissner et al., 2010). M4 also increase the secretion of matrix metalloproteinases (MMP7, MMP12), calcium-binding protein S100A8, and selected pro-inflammatory factors (IL-6, TNF-α), indicating that the M4 phenotype might be involved in degradation of the plaque fiber cap, allowing it to contribute to plaque instability and play a potential role in promoting atherosclerosis (Ethel et al., 2015).

Boyle et al. found a hemoglobin-responsive macrophage-Mhem, which is activated by heme, that overexpresses the scavenger receptor CD163 and upregulates HO-1 expression *via* CD163 in human bleeding plaques. Mhem can play a role in clearing iron and red blood cells through their phagocytosis and can be characterized by increased expression of IL-10 and apolipoprotein E (Boyle, 2012; Boyle et al., 2012). Boyle et al. also identified another new macrophage subset in hemorrhagic areas, known as HA-mac, which expresses high levels of CD163 but low levels of human leukocyte antigen (HLA) (Boyle et al., 2009). It was found that HA-mac is protective to blood vessels and shows anti-atherosclerotic and anti-inflammatory effects. This protective effect is achieved by sensing the hemoglobin-haptoglobin-complex through the CD163 receptor, increasing the hemoglobin gap, and reducing oxidative stress (Finn et al., 2012). Both Mhem and HA-mac have anti-inflammatory phenotypes and a decreased lipid uptake capacity, suggesting that they may possess similar effects.

In addition, macrophages also display strong phenotypic plasticity and differentiate into a variety of cells depending on the surrounding environment. Macrophages can aggregate in the endangium to swallow modified lipids and subsequently transform into foam cells, which promote the development of atherosclerosis (Otsuka et al., 2014). Macrophages also have the capacity to transdifferentiate into vascular wall cells (vascular smooth muscle cells, pericytes, and endothelial cells). Vascular endothelial growth factor can regulate the differentiation of macrophages into endothelial cells and promote angiogenesis (Yan et al., 2017). TGF-β can induce the differentiation of macrophages into smooth muscle-like cells through phosphorylation of Smad2 and Smad3 proteins (Ninomiya et al., 2006). Macrophage colony stimulating factor (M-CSF) or receptor activator for nuclear factor-κB ligand (RANKL) directly promotes the differentiation of macrophages toward osteoclast-like cells by triggering the TNF receptor associated factor 6 (TRAF6) signaling pathway, which affects the dynamic balance between osteogenesis and osteolysis (Kanazawa and Kudo, 2005). The differentiation of macrophages into multiple cell phenotypes affects the pathogenesis of various diseases to varying degrees.

## Role of Macrophages in Vascular Calcification

### Polarization Drift of Macrophages

**Figure 2:**
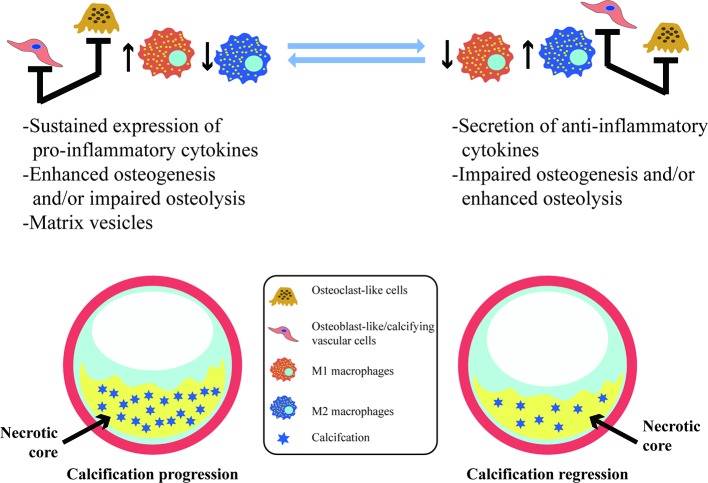
Vascular calcification and its association with macrophages. In vascular calcification progression, M1 sustained expression of pro-inflammatory cytokines and the release of calcifying matrix vesicles may promote calcification. Osteoblast-like/calcifying vascular cells predominating within lesions have the same pro-calcification effect. However, M2 macrophages contribute to resolving chronic inflammation, and osteoclast-like cells differentiated from macrophages are predominantly infiltrated with lesions may facilitate the regression of vascular calcification.

Vascular calcification is a dynamic process that is regulated and controlled by multiple factors. Macrophages are involved in almost all the stages of vascular calcification and play different roles in different subtypes in this process (**Figure 2**). M1 can directly release oncostatin M (OSM) to promote the differentiation of vascular smooth muscle cells (VSMCs) into osteoblastic phenotypes through the JAK3-STAT3 pathway (Zhang et al., 2017). The persistent state of chronic inflammation caused by M1 may also impair the normal development of VSMCs to osteoblasts, interfere with the maturation of osteoblasts, and, eventually, display scattered and fragmented calcification (Kraft et al., 2016). M2 can secrete anti-inflammatory factors as well as phagocytize necrotic fragments and apoptotic cells to prevent the formation of calcified nucleation sites (Braga et al., 2015). Ricardo et al. demonstrated that M2 can inhibit the osteogenic conversion of VSMCs using co-cultures of smooth muscle cells and macrophages to avoid direct intercellular contact. This inhibitory effect is related to increased adenosine triphosphate (ATP) secretion and pyrophosphoric acid (PPi) synthesis by M2 (via fatty acid β-oxidation) (Ricardo et al., 2016). Extracellular PPi is an endogenous inhibitor of vascular calcification *in vitro* and *in vivo* (Villa-Bellosta and Sorribas, 2013). PPi can be synthesized by extracellular ATP *via* ectonucleotide pyrophosphatase/phosphodiesterase 1 (ENPP1) *in vitro*, and deletion of ENPP1 can cause aortic calcification in mice (Johnson et al., 2005; Prosdocimo et al., 2009). In addition, PPi can be hydrolyzed to phosphoric acid (Pi) by tissue-nonspecific alkaline phosphatase (TNAP). Increased activity of TNAP contributes to ectopic calcification by disrupting the extracellular balance of PPi and Pi and generates adenosine to make up for reduced adenosine production in the genetic disease ACDC (Jin et al., 2016). Extracellular ATP can directly inhibit calcium-phosphate deposition and become hydrolyzed by ectonucleoside triphosphate diphosphohydrolase 1 (ENTPD1) to form Pi. It is worth noting that M2 increases the expression and activity of ENPP1, leading to an increase in the amount of PPi produced by hydrolysis of extracellular ATP. In contrast, the decreased expression of ENPP1 and increased expression of ENTPD1 in M1 results in decreased synthesis of PPi and increased hydrolysis of ATP (Ricardo et al., 2016). Moreover, M1 also produces high levels of TNF-α. TNF-α has been shown to enhance the TNAP activity of VSMC, which in turn, causes a subsequent reduction in the quantity of extracellular PPi and the formation of calcification *in vitro* (Lee et al., 2010).

When activated by different stimuli in different microenvironments, macrophages in different subsets can undergo polarity drift, i.e., macrophages in different subsets can be transformed into each other. Adamson S et al. found that M1 and M2 can be bio-transformed into each other under certain conditions, but macrophages polarization into M4 appears to be an irreversible process (Adamson and Leitinger, 2011). Cartilage oligomeric matrix protein (COMP) regulates macrophage polarity through integrin β3, a COMP-binding protein on the surface of macrophages. COMP deficiency can polarize macrophages into M1 phenotypes, i.e., they tend to be osteogenic phenotypes, and inhibit macrophages differentiation into M2 and osteoclast-like cells (Fu et al., 2016). In the apolipoprotein E- and COMP-deficient mice, increased calcification is observed in the artery. Macrophage-derived inflammatory factors (TLR4, IL-6), osteogenic factors (Wnt10b), and ROS production (iNOS) were also found to be markedly upregulated, but the expression of integrin β3 was decreased. Furthermore, the expression of inflammatory factors and osteogenic factors secreted by macrophage can be boosted by blocking COMP binding to integrin β3. ADAMTS-7 is the only COMP-degrading enzyme identified in blood vessels and is positively correlated with increased coronary and aortic calcification (Wang et al., 2009). Collectively, M1 and M2 have almost opposite effects in regulating the development of vascular calcification. Therefore, using the remarkable plasticity of macrophages to impel a polar shift in different subsets could offer a new breakthrough for calcification regression. Investigators have also found that protocatechuic acid (PCA) can inhibit macrophage polarization into M1 through the PI3K −Akt−NF-κB−SOCS1 pathway and enhance M2 phenotype polarization *via* the STAT6−PPARγ pathway (Liu et al., 2019). Curcumin has also been demonstrated to have similar effects *via* inhibition of M1 and promotion of M2 macrophage polarization as a novel anti-inflammatory agent (Karuppagounder et al., 2016). Macrophage polarization is a multi-factor process, and hence, investigation of which signaling pathway is actually involved in the process has important implications for precise adjustment of the mutual transformation between M1 and M2 and prevention and treatment of vascular calcification.

### Functional Substances Secreted by Macrophages

Recently, new molecular imaging studies have confirmed that inflammation is closely related to atherosclerotic calcification and may occur prior to calcification. Macrophages can secrete various inflammatory factors (such as TNF-α, IL-1β, IL-6), which not only promote differentiation of vascular wall cells into chondrocytes and osteoblasts but also stimulate the production of oxygen free radicals and the expression of bone morphogenetic protein (Byon et al., 2011; Martinet et al., 2012). These pathways are all involved in the formation of vascular calcification. IL-1β can be used as a potent predictor of incident cardiovascular disease, and the high calcium burden of the coronary artery is positively correlated with the level of serum IL-1β. The main mechanism of IL-1β production is that Rac1 in macrophages promotes the activation of NF-κB and the production of ROS through NADPH oxidase (Hordijk, 2006). Nicolle et al. found that the absence of Rac2 in macrophages can increase the activity of Rac1, leading to increased expression of IL-1β, and further promoting the development of calcification. In terms of regulating the expression of IL-1 β, an antagonistic relationship exists between Rac2 and Rac1. The absence of Rac2 leads only to increased Rac1 activity but has no effect on the expression of Rac1. The mechanisms behind this antagonistic relationship may be the competition of common guanine nucleotide exchange factors (Ceneri et al., 2017). Also of note, the clinical applicability of current biomarkers is more frequently used as a predictor of cardiovascular risk than a guide to therapy. Hence, it will be challenging and difficult to define specific biomarkers and establish repeatable and accurate methods to evaluate the effects of new therapeutic interventions in vascular calcification. Recently, Abigail et al. found that although statins have been widely developed as hypolipidemic drugs, they can promote atherosclerotic calcification by relieving the inhibitory effect of RhoGDI (Rho GDP-dissociation inhibitor) on the Rac1-IL-1βsignaling axis in macrophages. Supplementing the isoprenaline precursor can offset the pro-calcified effects of statins to a certain extent (Healy et al., 2020). The increase in calcium deposition caused by statins may be associated with plaque stability. To further dissect the potential molecular mechanisms of statins in plaque vulnerability, a standardized small animal model for plaque rupture is highly warranted. In addition, using Rac (small GTPase and key signal transducer of inflammatory cells) as a potential therapeutic target to inhibit vascular calcification might be an important direction for future research.

In addition, the osteogenic genes released by macrophages, such as runt-related transcription factor 2 (Runx2), TNAP, osteoprotegerin (OPN), and bone morphogenetic protein 2 (BMP-2), can also promote the osteogenic process. A macrophage conditioned medium that leads to the overexpression of Wnt10b, BMP-6, and SPHK1 (catalyzing sphingosine phosphorylation to form sphingosine-1-phosphate) can stimulate migration and differentiation of human mesenchymal stem cells (MSCs) into osteoblast lines (Shao et al., 2007). BMP-2 can promote the phenotypic transformation of human bone marrow MSCs into osteoblasts and selectively act on type I or type II receptors to upregulate Runx2 expression (Donoso et al., 2015). Runx2 is a key transcription factor in the process of osteoblastic differentiation and chondrocyte maturation, as well as a key regulator in the process of chondrogenic differentiation in vascular wall cells. Runx2 can regulate the expression of multiple osteogenic marker genes (alkaline phosphatase, collagen type I, osteosalsialin, osteopontin, etc.) to promote vascular calcification (Sheen et al., 2015). Recently, the osteogenic genes expressed by macrophages have been intensively studied by many investigators. Dube et al. demonstrated that selective deletion of TRPC3 (transient potential classical receptor 3, a nonselective calcium channel) in macrophages can reduce apoptosis of macrophages induced by endoplasmic reticulum stress and also attenuate the expression of BMP2 and Runx2 to reduce calcification in advanced atherosclerotic plaques (Dube et al., 2018). Nakano T also made use of the Delta-like 4 (DLL4) inhibitor, which can suppress the expression of BMP2 and Runx2 in macrophages to prevent the formation of vascular calcification *via* the Notch signaling pathway (Nakano et al., 2016). Teniposid [a Topo II (DNA topoisomerase II) inhibitor] regulates the p53-(miR-203-3p)-BMP2 signaling pathway to attenuate the expression of BMP2 and Runx2 and has anti-calcification effects (Liu et al., 2018). Poly [ADP-ribose] polymerase 1 (PARP-1) deficiency can reduce the expression of Runx2 by inactivating STAT1 and also accelerate the pace of M1 polarization into M2, which prevents the progression of diabetes-induced atherosclerotic calcification (Li et al., 2020). Osteogenic proteins, which are mediated by multiple signaling pathways, have great potential in regulating the development of vascular calcification.

MVs secreted by macrophages are the nucleation sites of calcium phosphate crystals. In the early stage of vascular calcification, marked accumulation of macrophages can be readily observed. Macrophages release large amounts of calcifying MVs containing the phosphatidylserine-annexin V-S100A9 complex (Wang et al., 2016). Sophie et al. confirmed that on the surface of a macrophage-derived MV membrane, phosphatidylserine can form complexes with annexin V and S100A9, which can promote the entrance of calcium ion into vesicles. Calcium ions and phosphate ions entering vesicles through phosphate channels accumulate in the form of a calcium phosphorus complex on the inner side of the membrane and form the initial hydroxyapatite crystals. Subsequently, the hydroxyapatite crystals continued to grow until the membrane ruptures, bind with the extracellular matrix as the core of the calcium salt crystal, and constantly grow to form calcified nodules. During this process, alkaline phosphatase on the vesicle membrane continuously supplies phosphorus ions by decomposing ATP/ADP and pyrophosphate (New et al., 2013; Jing et al., 2019). MVs also contain micronucleus glyconucleic acids, which can promote the deposition of calcium orthophosphate, transformation of calcium orthophosphate into amorphous calcium phosphate and development into more crystalline structures, such as hydroxyapatite (Zazzeroni et al., 2018). Compared with osteoclast-derived MVs, monocytes-derived MVs convey different signals to MSCs and upregulated the expression of MMPs at the same time. MVs from activated monocytes facilitate osteogenic differentiation of MSCs and upregulate the secretion of multiple factors by MSCs to play a immunosuppressive role (Gebraad et al., 2018). Matrix metalloproteinases (MMPs) secreted by macrophages are a family of zinc-dependent endopeptidases that play crucial roles in several pathological processes. MMP-9 enhances macrophage infiltration to the lesion site and digestion of extracellular matrix. Increased MMP-9 can also hydrolyze elastin, and elastin-derived peptides contribute to the osteogenic differentiation of VSMCs (Chen et al., 2020). Furthermore, whether increased MMP-9 expression in turn promotes the release of pro-calcified MVs still requires further investigation. Moreover, macrophage-derived MMP-10 promotes the expression of BMP2 and Runx2 and facilitates the development of vascular calcification (Purroy et al., 2018). In summary, bioactive substances originated from macrophages are worthy of additional in-depth research prior to clearing its complex mechanisms on vascular calcification.

### Phenotypic Transformation of Macrophages

Vascular calcification is a dynamic process that represents a balance between osteogenesis and osteolysis. Once the balance is broken, pathological calcification easily occurs (**Figure 2**). Macrophages accelerate calcification by promoting the transformation of vascular wall cells into osteoblastic phenotypes and also promote the remodeling and regression of vascular calcification by transforming into osteoblast-like cells that secrete acid, which dissolves hydroxyapatite crystals, and protease, which degrades bone organic matrix. TNF-α plus CaPO4 stimulates the transformation of macrophages into osteoclast-like cells. However, the mechanism is different from the traditional RANK-RANKL pathway and does not trigger the TRAF6 signaling pathway. In contrast, the mRNA expression level of TRAF2 is significantly increased in this process, and inhibition of TRAF2 can significantly suppress the osteoclastogenesis induced by TNF-α plus CaPO4 (Yuichiro et al., 2016).

Promotion of the differentiation of macrophages into osteoclast-like cells is conducive to the regression of vascular calcification. Conversely, inhibition of osteoclast differentiation or dysfunction of osteoclastic bone resorption activity may be related to the emergence and persistence of vascular calcification. Giulia et al. found that although the expression of the osteoclast marker CA2 was enhanced after macrophages were treated with IL-4, the expression and activity of CTSK (Cathepsin K, a major enzyme involved in osteoclastic bone resorption activity) was decreased (CChinetti-Gbaguidi et al., 2017). The mechanism might be summarized as impaired phosphorylation of ERK1/2 after IL-4 stimulation of macrophages, resulting in the reduction of c-fos activity as a activator of nuclear factor of activated T cells c1 (NFATc1) expression or reduced expression of NFATc1 by upregulating the promoter H3K27me3 at the epigenetic level. This process impairs the expression of key factors for osteoclast-like bone resorption activity of macrophages, including CTSK, leading to osteoclastic bone resorption dysfunction. A reduction of macrophage-derived osteoclast-like cells could be obviously observed in patients with uremia complicated with vascular calcification, which further indicates that osteoclast-like cells play a key role in the process of suppressing vascular calcification (Massy et al., 2008).

Vascular smooth muscle cells can be transformed into macrophages and MSCs under certain conditions. Conditional knockout of VSMC specific Krüppel-like factor 4 (KLF4) can reduce the number of SMC-derived macrophages and MSCs (Shankman et al., 2015). The question of whether macrophages are equally capable of differentiating into SMC has caused a hot debate. Inflammatory factors play an important role in the transdifferentiation of macrophages into smooth muscle cell-like cells and Kozo et al. experimentally confirmed the strongest power of transforming growth factor β1. Macrophages cultured with 10% fetal bovine serum containing TGF β1 can increase the expression of SMC markers (α-SM actin, calponin, and SMemb) in a dose-dependent manner within a certain concentration range. This process is partially dependent on the phosphorylation of Smad2 and Smad3 (Ninomiya et al., 2006). Smooth muscle-like cells actively participate in the regulation of vascular calcification. Under various stimuli, such as a high glucose environment and oxidative stress, smooth muscle-like cells can switch from a contractile morphology to a synthetic phenotype and, finally, to an osteoblast-like phenotype to promote vascular calcification (Chen et al., 2017). In recent year, interest has grown in the intrinsic calcification capacity of pericytes (PC), which are versatile glia cells in the vascular wall. Studies have confirmed that PC have an active phagocytosis capacity and express macrophage-specific antigen (CD68), showing an unignorable association between the two (Cochain and Zernecke, 2015). The ability of PC to differentiate toward osteogenic and chondrogenic phenotypes has been proven by animal experiments and *in vitro* cellular experiments, and IL-6 may play a crucial role in aberrant differentiation of PC (Leszczynska et al., 2016). In a human femoral artery specimen with osteoid metaplasia, Davaine et al. found notably increased CD146-positive and NG2-positive pericytes, accompanied by increased expression of OPG. *In vitro* experiments further showed that the OPG/RANKL/RANK pathway mediates PC differentiate into osteoblast-like cell and suppress CD14-positive cells differentiation into osteoclast-like cells, finally exacerbating vascular calcification (Davaine et al., 2016). The glucocorticoid dexamethasone has been verified to promote PC differentiation into an osteoblast-like phenotype and to aggravate calcification by downregulating inhibitors of calcification, such as MGP and OPN (Kirton et al., 2006). In summary, these findings suggest that phenotypic transformation of macrophages affects the progression and regression of vascular calcification to varying degrees, and promoting the differentiation of macrophages into beneficial phenotypes might become feasible for treatment of vascular calcification.

## Conclusion and Future Directions

Vascular calcification is a complex process that involves multiple factors. Disorder of the calcium and phosphorus metabolism balance, the imbalance between mineralization inducers and inhibitors, and the imbalance between osteoclast‐mediated bone resorption and osteoblast‐induced bone formation all have significant impacts on the formation and regression of vascular calcification. Macrophages are closely related to vascular calcification. A research boom has occurred based on the role of macrophages in the process of vascular calcification. The mechanisms of how macrophages can intervene in the progression and regression of vascular calcification have become increasingly clear. However, the phenotypic plasticity and functional heterogeneity of macrophages also lead to their pleiotropic effects. The question of how to precisely regulate the differentiation of macrophages into a phenotype in a manner that is conducive to calcification regression may become an urgent problem to be solved. Therefore, it is significant in theory and in practice to further identify and characterize macrophage subsets using single-cell technology. Understandably, additional questions currently exist relative to these potential roles of macrophages in pathological vascular biology: Can we achieve the treatment purpose of vascular calcification by targeting and regulating the M1 shift into M2 or promoting macrophages around calcified areas to differentiate into functional osteoclast-like cells while limiting undesired macrophage functions? Which other mechanisms might participate in the inhibition of bone resorption capacity of macrophage-derived osteoclasts surrounding calcium deposits? What mechanisms initiate and mediate the release/enrichment of macrophage-derived MVs? By which mechanisms do macrophages and their active substances interact and communicate with vascular wall cells? The current research has not yet supplied a clear-cut answers to these questions. Due to the existence of the bone-vascular axis and calcification paradox, it is necessary to further explore whether the reversal of vascular calcification by targeting macrophages could lead to bone loss in bone tissue. Many anti-calcification drugs have been restricted from clinical application due to various potential side effects. Active development of drugs such as teniposide, which can effectively inhibit vascular calcification in a tissue-dependent manner but had no effect on bone structure, is expected to be a boon for the treatment of vascular calcification. In conclusion, further exploration of the potential molecular mechanism of how macrophages regulate the progression and regression of vascular calcification is expected to reveal a new entry point for the prevention and treatment of vascular calcification, with far-reaching implications.

## Author Contributions

ZW conceived and designed review; YL, ZS, LZ and LJ wrote and revised manuscript; YL, ZS, LZ, JY, CS, LJ, LL and ZW approved final version of manuscript.

## Funding

This work was supported by the foundations as follows: the National Natural Science Foundation of China (Grant Nos:81770450, 81370408, 81670405); the related Foundation of Jiangsu Province (WSN-044, LGY2018092,QNRC2016836); the Project Funded by the Priority Academic Program Development of Jiangsu Higher Education Institutions; Postgraduate Research & Practice Innovation Program of Jiangsu Province (SJKY19_2585); Zhenjiang Cardiovascular Clinical Research Center Project (SS2018008).

## Conflict of Interest

The authors declare that the research was conducted in the absence of any commercial or financial relationships that could be construed as a potential conflict of interest.

## Abbreviations

TRAP, tartrate resistant acid phosphate; YSDM, yolk sac derived macrophages; BMDM, bone marrow derived macrophages; M1, the classical activated macrophage; M2, the alternative activated macrophage; INF-γ, interferon-γ; IL, interleukin; TGF, tumor growth factor; CXCL10, CXC chemokines ligand 10; iNOS, nitric oxide synthetase; COX2, cyclooxygenase 2; Arg1, arginase 1; TLR, Toll-like receptor; ROS, reactive oxygen species; VEGF, vascular endothelial growth factor; HO-1, hemeoxygenase-1; MMP, matrix metalloproteinases; M-CSF, macrophage colony stimulating factor; RANKL, receptor activator for nuclear factor-κB ligand; RANK, receptor activator for nuclear factor-κB; TRAF6, TNF receptor associated factor 6; OSM, oncostatin M; VSMCs, vascular smooth muscle cells; Runx2, runt-related transcription factor 2; ATP, adenosine triphosphate; ADP, adenosine diphosphate; PPi, pyrophosphoric acid; ENPP1, ectonucleotide pyrophosphatase/phosphodiesterase 1; Pi, phosphoric acid; TNAP, tissue-nonspecific alkaline phosphatase; ACDC, arterial calcification due to deficiency of CD73; ENTPD1, ectonucleoside triphosphate diphosphohydrolase 1; COMP, cartilage oligomeric matrix protein; NF-κB, nuclear-factor-κB; PI3K, phosphoinositide 3-kinase; SOCS1, suppressor of cytokine signaling 1; STAT6, signal transducers and activators of transcription 6; PPARγ, peroxisome proliferator activated receptor γ; NADPH, nicotinamide adenine dinucleotide phosphate; BMP, bone morphogenetic protein; SPHK1, sphingosine kinase 1; TRPC3, transient potential classical receptor 3; CTSK, cathepsin K; ERK, extracellular signal-regulated-kinase; NFATc1, nuclear factor of activated T cells c1; KLF4, specific Krüppel-like factor 4; MGP, matrix gla protein; OPG, osteoprotegerin; OPN, osteopontin.
